# Protective Effects of *Bacillus coagulans* JA845 against D-Galactose/AlCl_3_-Induced Cognitive Decline, Oxidative Stress and Neuroinflammation

**DOI:** 10.4014/jmb.2111.11031

**Published:** 2021-12-22

**Authors:** Xinping Song, Zijian Zhao, Yujuan Zhao, Qing Jin, Shengyu Li

**Affiliations:** 1 College of Agriculture, Yanbian University, Yanji 133002, P.R. China; 2 Institute of Agro-food Technology, Jilin Academy of Agricultural Sciences, Changchun 130033, P.R. China

**Keywords:** *Bacillus coagulans*, cognitive decline, oxidative stress, neuroinflammation

## Abstract

Recently, the efficacy of probiotics in treatment of neurodegenerative disorders has been reported in animal and clinical studies. Here, we assessed the effects of *Bacillus coagulans* JA845 in counteracting the symptoms of D-galactose (D-gal)/AlCl_3_-induced Alzheimer&rsquo;s disease (AD) in a mice model through behavioral test, histological assessment and biochemical analysis. Ten weeks of pre-treatment with *B. coagulans* JA845 prevented cognitive decline, attenuated hippocampal lesion and protected neuronal integrity, which demonstrated the neuroprotective features of *B. coagulans* JA845 in vivo. We also found that supplementation of *B. coagulans* JA845 alleviated amyloid-beta deposits and hyperphosphorylated tau in hippocampus of D-gal/AlCl_3_-induced AD model mice. Furthermore, *B. coagulans* JA845 administration attenuated oxidative stress and decreased serum concentration of inflammatory cytokines by regulating the Nrf2/HO-1 and MyD88/TRAF6/NF-&kappa;B pathway. Our results demonstrated for the first time that *B. coagulans* has the potential to help prevent cognitive decline and might be a novel therapeutic approach for the treatment of neurodegenerative diseases.

## Introduction

Alzheimer’s disease (AD) is a prevalent, chronic and irreversible neurodegenerative disease that contributes to gradual cognitive decline, behavioral abnormities and brain lesions in the elderly (>65 years of age). The representative pathologic hallmarks of AD include extracellular senile plaques composed of neurotoxic amyloid-beta (Aβ) peptides and hyperphosphorylated tau protein leading to neurofibrillary tangles [1]. Although the Aβ and tau hypothesis is widely accepted as AD pathogenesis, it has been reported that oxidative stress, inflammation and gut dysbiosis also have a causal role in AD pathogenesis [2-4]. Hence, treatment or prevention of AD considering reduction of oxidative stress or inflammation may serve as a better therapeutic strategy.

The beneficial use of probiotics can balance intestinal microecology by inhibiting the proliferation of pathogens and increasing the number of beneficial bacteria, which have beneficial effects on the immune response, metabolism and brain function [5]. Emerging evidence suggests that consumption of probiotics has great potential in treatment or prevention of central nervous system-related diseases. It has been reported that *Bifdobacterium breve* strain A1 prevented cognitive dysfunction and reduced neuronal inflammation by modulating immune-reactive genes in an Aβ-induced AD model mice [6]. In addition, the intervention of lactic acid bacteria and bifidobacteria significantly alleviated oxidative damage via regulating the SIRT1 pathway in a triple-transgenic AD model mice [7]. To date, a few strains of probiotics, most of which are traditional probiotics including *Lactobacillus* spp. and *Bifidobacterium* spp., have been applied in preventing and treating AD.

*Bacillus coagulans* is characterized by spore production and has been widely applied in the food, pharmaceutical and breeding industries. *Bacillus coagulans* has improved survival and stability in terms of production, preservation and administration compared with traditional probiotics, such as *lactobacillus* and *bifidobacteria*. which may be attributed to the property of spore production [8]. Thus, it is a suitable candidate for application in functional foods and pharmaceutical formulations. Animal and preclinical studies have shown that *Bacillus coagulans* has health-promoting effects on gastrointestinal tract diseases, such as irritable bowel syndrome [9], antibiotic-related diarrhea [10], inflammatory bowel disease [11] and constipation [12]. Recently, the efficacy of *B. coagulans* MTCC 5856 in the treatment of patients suffering from irritable bowel syndrome with major depressive disorder was reported in a clinical study [13]. Additionally, multi-strain probiotics containing *B. coagulans* were effective in relieving the pressure associated with examination [14]. These findings show great potential of treating psychiatric and neurological diseases through consumption of *B. coagulans*. Nevertheless, precious few studies have been available in terms of the beneficial effects of *B. coagulans* on central nervous system-related diseases. In the present study we explored the protective effects of *B. coagulans*, with a particular focus on the improvement of AD-like symptomatology in D-galactose (D-gal)/AlCl_3_-induced model mice. Specifically, we explored the beneficial effects of *B. coagulans* JA845 (isolated from Chinese traditional fermented pickle) in improving cognitive decline, oxidative injury and neuroinflammation related to AD. Our results revealed that *B. coagulans* JA845 could prevent cognitive decline and brain lesion, and attenuate oxidative damage and neuroinflammation by regulating the MyD88/TRAF6/NF-κB and Nrf2/HO-1 pathway.

## Materials and Methods

### Culture of Probiotics

*Bacillus coagulans* JA845 (JA845) was isolated from Chinese traditional fermented pickle and preserved at the China General Microbiological Culture Collection Center (No.19576; CGMCC, China). *B. coagulans* JA845 was inoculated in Luria-Bertani liquid medium containing glucose, yeast extract and salt, and cultured at 180 ×*g* at 50°C for 10 h. Then, *B. coagulans* JA845 was resuspended in saline and adjusted to 1 × 10^9^ colony-forming units (CFU)/ml for experimental use.

### Animals and Treatment

Male ICR mice weighing 20-23 g were provided by Yisi Laboratory Animal Technology Co., Ltd. (Changchun, China) and raised in a ventilated cage under a controlled environment (temperature, 22 ± 1°C; 12 h light/dark cycle). The animal studies were approved by the Committee for Laboratory Animal Care and Use at Jilin Academy of Agricultural Sciences (SCXK2020-0001) and performed in accordance with the guidelines of the same. The 24 mice were randomly separated into three groups, 8 mice in each group (*n* = 8). The probiotics pre-treatment lasted for 10 weeks. The control group (control) received saline at 10 ml/kg BW/day. The D-gal+AlCl_3_-induced AD model model group (model) intraperitoneally received D-galactose (120 mg/kg BW/day) and was intragastrically administrated with AlCl_3_ (30 mg/kg BW/day), followed by oral saline treatment at 10 ml/kg BW/day after 2 h. The JA845 group (JA845) intraperitoneally received D-galactose (120 mg/kg BW/day) and was intragastrically administrated with AlCl_3_ (30 mg/kg BW/day), followed by oral administration of *B. coagulans* JA845 (1 × 10^9^ CFU/day/mouse) after 2 h of D-gal+AlCl_3_ intervention. Behavioral tests were performed after 10 weeks. The mice were sacrificed after the behavioral test was completed, and then serum samples and brains were collected and stored at −70°C for subsequent analyses.

### Behavioral Tests

The Morris water maze test (MWM) was performed after JA845 intervention for 10 weeks to assess spatial learning and memory of the mice, as previously described [15]. The maze is comprised of a circular cistern (diameter: 120 cm; depth: 50 cm) filled with water (30 cm high), with a platform (diameter: 10 cm) submerged 1 cm below the water surface. The training trials were performed for 5 days, and mice were placed in the cistern from the starting location of four different quadrants and tasked to locate the submerged platform within 60 s. In case the mouse could not find the hidden platform, the experimenter guided the animal to the platform, allowing it to stay there for 10 s to consolidate memory. The probe trial was performed with platform removed from the cistern within 60 s. The infrared tracking equipment (ZH-Morris, Anhui Zhenghua Biological Instrument Equipment, China) recorded escape latency, platform-crossing time, and swimming trajectory.

The short-period memory of mice was evaluated by the step-down test. Each mouse was left in the reaction box with a raised platform and the electrified floor, adapted to the environment with 3 min before the testing phase. Then, each mouse was placed on the raised platform and subsequently given an electric shock (0.5 mA) for 5 min. The equipment recorded the error numbers and latency on the platform for the test duration.

### Hematoxylin and Eosin (H&E) Staining

The hippocampal pathological alterations of mice were detected by H&E staining after completion of the behavioral test. The hippocampus of each mouse was separated from the brain, fixed with 4 % paraformaldehyde for 24 h, then embedded in paraffin. Hippocampal tissue sections were sliced at 3 μm by apparatus (Leica, China), then stained with H&E. The histological changes of hippocampus were detected using light microscopy (Olympus, Japan) at 400 × magnification.

### Immunohistochemistry

The hippocampal sections were deparaffinized and rehydrated for immunohistochemical staining of Aβ and p-tau (Ser 404). The sections were treated with citric acid antigenic repair buffer for antigen retrieval in a microwave oven, and washed with phosphate-buffered saline (PBS) (pH 7.4) three times for 5 min. Then, hippocampal sections were incubated with 3% hydrogen peroxide for 25 min away from light, and then washed with PBS. Subsequently, hippocampal slices were blocked with 3% bovine serum albumin (BSA) for 30 min, incubated with primary and secondary antibody, and checked for development of immunoreactions by diaminobenzidine chromogen staining. The sections were analysed using light microscopy.

### Biochemical Analyses

Blood was collected into a tube and centrifuged at 2,400 ×*g* at 4°C for 10 min for biochemical analyses. The serum levels of superoxide dismutase (SOD), malondialdehyde (MDA), nitric oxide (NO), catalase (CAT), glutathione (GSH), glutathione peroxidase (GSH-Px), tumor necrosis factor-α (TNF-α), interleukin-6 (IL-6) and interleukin-1β (IL-1β) were detected using commercial ELISA kits (Meibiao, China).

### Western Blot Analysis

The total protein was extracted from brain tissues, then homogenized in RIPA lysis buffer including protease inhibitor (Solarbio, China) and phosphatase inhibitor (Solarbio, China) for 30 min on ice. Protein of supernatant was collected after centrifugation for 15 min (13,600 ×*g*, 4°C). Total protein was detected using a bicinchoninic acid (BCA) kit (Thermo Scientific, USA). Brain lysates were electrophoresed on 10% SDS-PAGE, transferred to nitrocellulose membrane, then blocked with tris-buffered saline with Tween 20 (TBST) solution containing 3%BSA. Membranes were probed with primary antibodies for MyD88 (Abcam, USA), TRAF6 (Abcam, USA), NF-κB p65 (Abcam), Nrf2 (Bioss, China), HO-1 (Bioss, China), GAPDH (Abcam) and β-actin (Abcam) overnight at 4°C, and washed with TBST 3 times for 10 min. Membranes were treated with HRP-conjugated anti-rabbit antibody (Bioss, China) the next day. Protein bands were analyzed using enhanced chemiluminescence and calculated using ImageJ software.

### Statistical Analysis

Statistical analysis was carried out using SPSS 22.0 software. All data were expressed as mean±SD. The results were analyzed by Shapiro-Wilk normality test, followed by normal distribution performed using one-way analysis of variance followed by Dunnett’s post hoc test, or Kruskal-Wallis test followed by Dunnett’s post hoc test. Significant difference was accepted with a value of *p* < 0.05.

## Results

### *B. coagulans* JA845 Alleviated Cognitive Decline in D-gal/AlCl_3_-Induced AD Model Mice

**Fig. 1:**
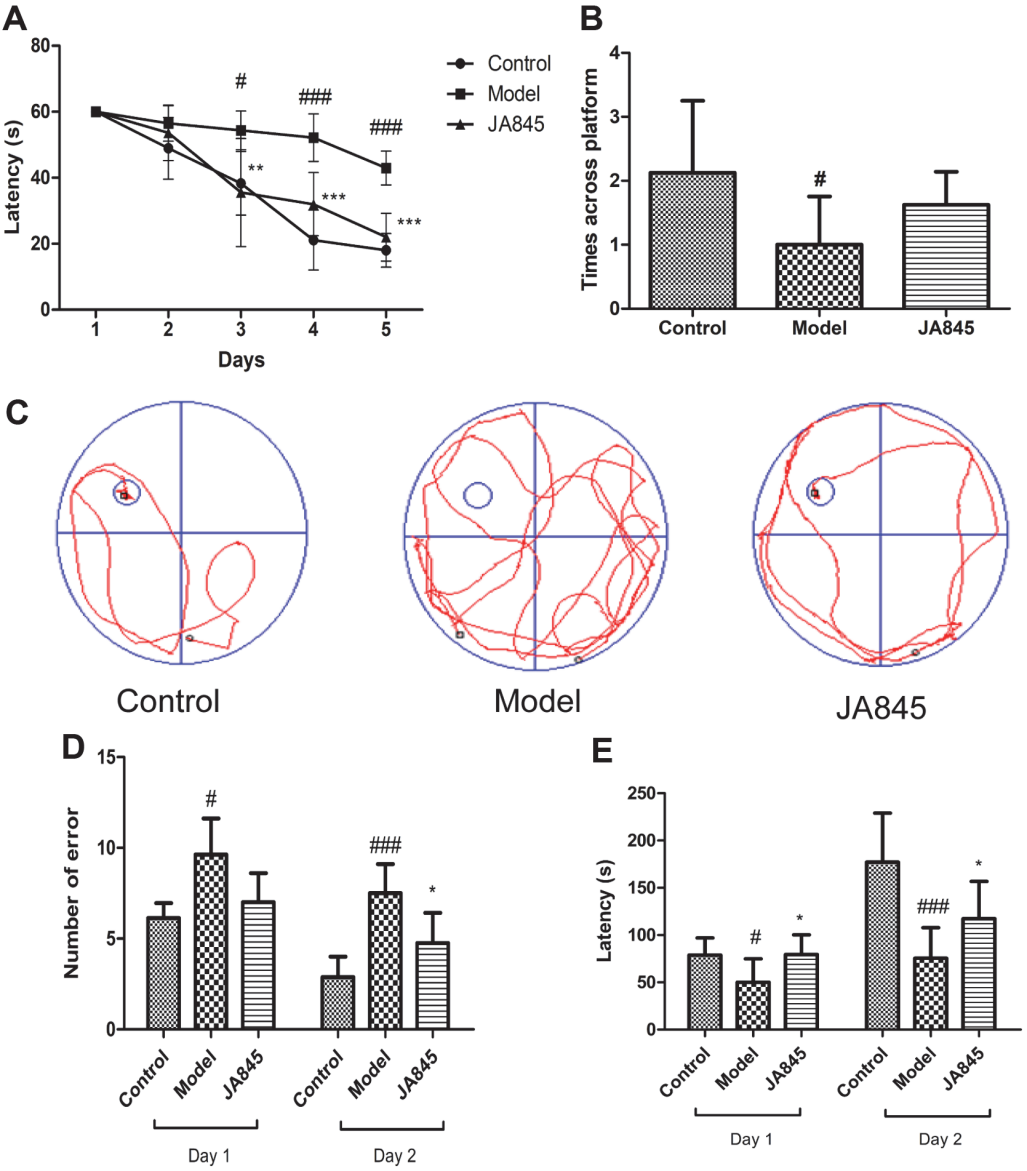
Effect of *B. coagulans* JA845 on cognitive function in D-gal/AlCl_3_-induced AD model mice. (**A**) Latency in Morris water maze test. (**B**) The representative infrared trajectory on the last day of place navigation test. (**C**) Platformcrossing times. (**D**) Error numbers in step-down test. (**E**) Latency in step-down test. *p < 0.05, **p < 0.01, ***p < 0.001, compared with model group. #*p* < 0.05, ##*p* < 0.01, ###*p* < 0.001, compared with control group.

Figure 1 shows the spatial learning and memory performance of the three groups by MWM test and step-down test. *B. coagulans* JA845 significantly reduced escape latency on training days, especially on the fourth and fifth day (*p* < 0.001) compared with the D-gal/AlCl_3_-induced AD model mice (Fig. 1A). Indeed, JA845 obviously shortened the swimming track to the platform, and the representative infrared trajectory of AD model mice was more disordered than that of the JA845 group, suggesting beneficial effect of learning memory after usage of JA845 (Fig. 1B). JA845 group increased platform-crossing times in the probe trial, but no significant differences were found between JA845 group and AD model group (Fig. 1C). Furthermore, JA845 pre-treatment showed fewer error numbers and longer retention latency compared with AD model group in training and testing context of step-down test (Figs. 1D, 1E), implying the beneficial effect of short-term working memory after JA845 pre-treatment. Behavioral tests indicated that *B. coagulans* JA845 prevented cognitive deficits in D-gal/AlCl_3_-induced AD model mice.

### Effect of *B. coagulans* JA845 on Hippocampal Histological Changes, Aβ Accumulation and Tau Phosphorylation in D-gal/AlCl_3_-Induced AD Model Mice

**Fig. 2:**
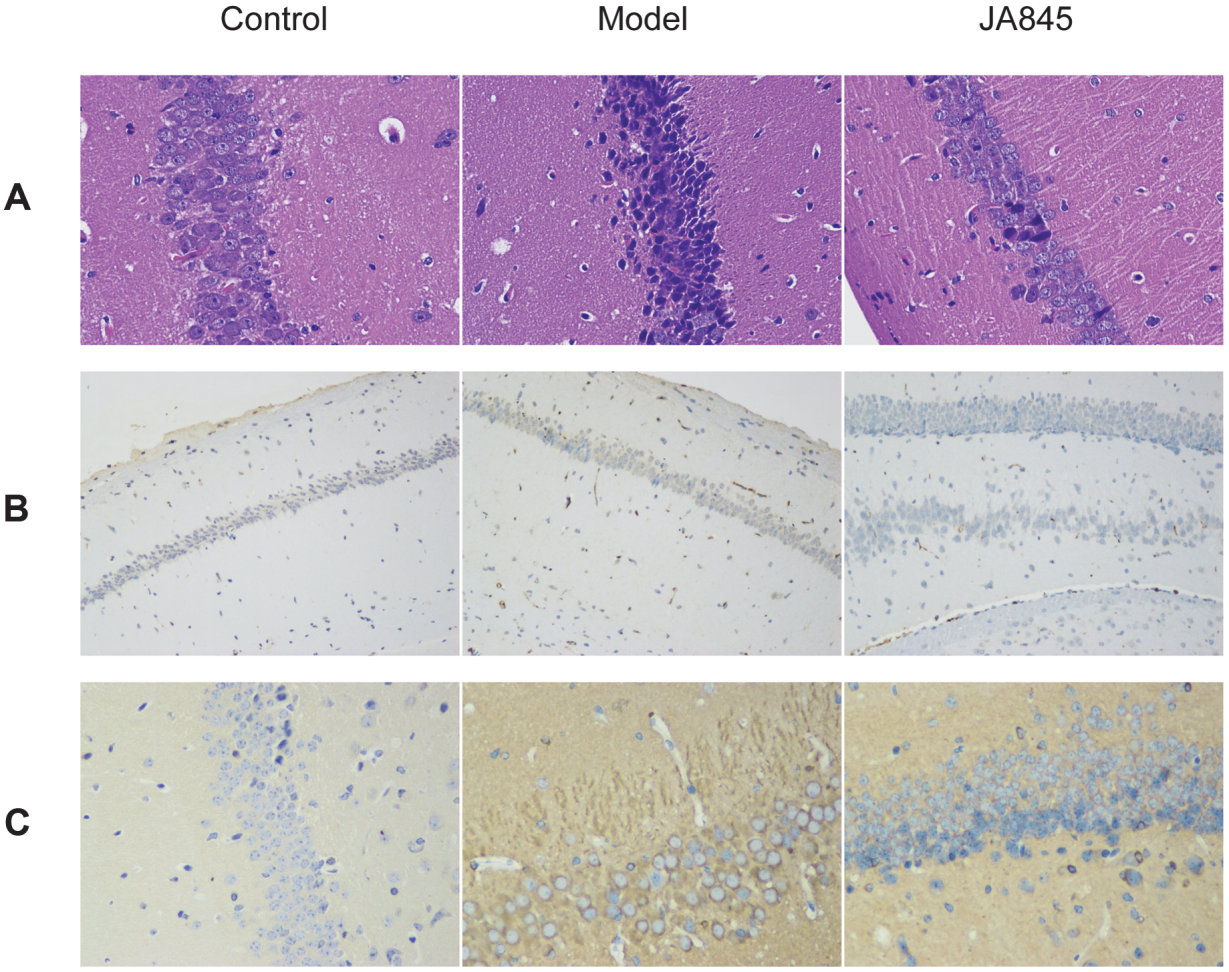
Effect of *B. coagulans* JA845 on hippocampus histology, Aβ deposition and tau pathology in D-gal/AlCl_3_-induced AD model mice. (**A**) HE staining of hippocampus (400 × magnification). (**B**) Expression of Aβ in hippocampus by immunohistochemical staining. (**C**) Expression of p-tau (Ser 404) in hippocampus by immunohistochemical staining.

As shown in Fig. 2, the effects of *B. coagulans* JA845 on histopathological changes of hippocampus in D-gal/AlCl_3_-induced AD model mice were explored though H&E staining. The shrunken neuronal cells contributed to the excessive clearance among neurons, and the broken pyramidal layer, small pyknotic nuclei with the uneven distribution as well as deeper staining were obviously found in AD group. Conversely, JA845 significantly reduced loss of neurons and the cells were arranged in an orderly fashion, and this pathological feature was in close proximity to the control group. The histological results suggested that the chronic administration of D-gal and AlCl_3_ combination resulted in hippocampal histological changes, which could be reversed by *B. coagulans* JA845.

To investigate Aβ plaques and tau pathology in response to *B. coagulans* JA845, immunohistochemical staining was performed on hippocampal tissues. The extracellular Aβ plaques were obviously found in hippocampal tissues of AD model mice; meanwhile, JA845 reduced the accumulation of extracellular Aβ compared with AD mice. Furthermore, JA845 administration ameliorated tau phosphorylation at Ser404 in D-gal/AlCl_3_-induced AD model mice. These results revealed that the beneficial effects of *B. coagulans* JA845 in reducing Aβ accumulation and tau phosphorylation, which are pathological features of the AD brain.

### Effect of *B. coagulans* JA845 on Oxidative Stress and Inflammatory Response in D-gal/AlCl_3_-Induced AD Model Mice

**Fig. 3:**
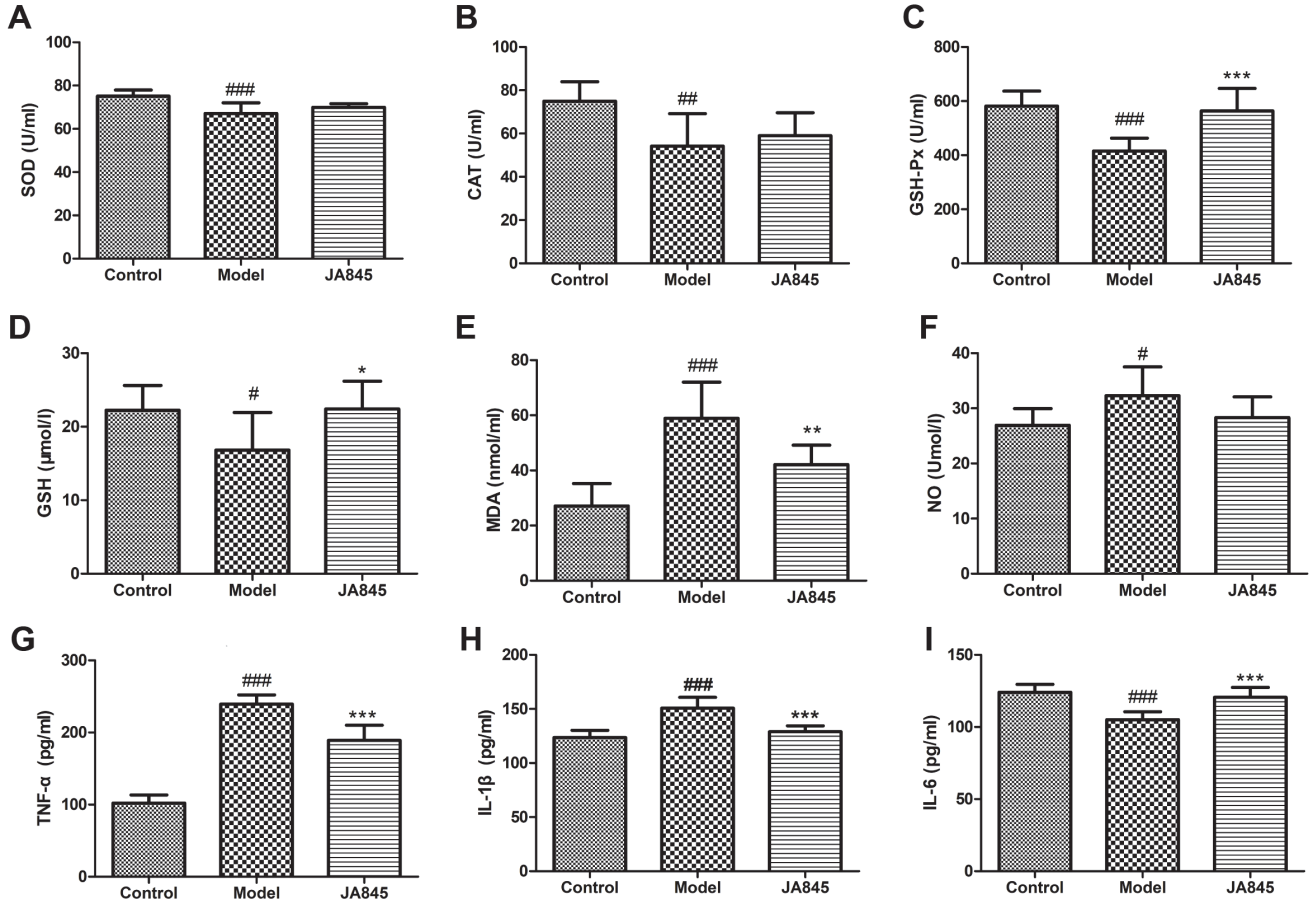
*B. coagulans* JA845 decreased oxidative stress and inflammatory response in serum of D-gal/AlCl_3_-induced AD model mice. (**A**) SOD activity, (**B**) CAT activity, (**C**) GSH-Px activity, (**D**) GSH level, (**E**) MDA level, (**F**) NO level, (**G**) TNF-α level, (**H**) IL1b level, (**I**) IL-6 level. *p < 0.05, **p < 0.01, ***p < 0.001, compared with model group. #*p* < 0.05, ##*p* < 0.01, ###*p* < 0.001, compared with control group.

We determined the serum concentrations of GSH, MDA, NO, SOD, CAT and GSH-Px to examine whether JA845 had anti-oxidative effects in AD model mice. As shown in Fig. 3, no significant differences in SOD and CAT activities were found between JA845 group and AD model group. Significantly increased GSH-Px activity was observed in JA845 group compared with AD model group (*p* < 0.001). Moreover, the serum concentration of GSH in JA845 group was significantly higher than that of the AD model group (*p* < 0.05), while lower levels of MDA and NO were found in JA845 group.

We also measured inflammatory cytokines including TNF-α, IL-6, and IL-1β to assess the anti-inflammatory effects of JA845. Our results showed significant differences in serum levels of inflammatory cytokines between JA845 group and AD model group. As shown in Figs. 3, the concentrations of inflammatory cytokines including TNF-α and IL-1β were significantly decreased in JA845 group compared with AD model mice, while the concentrations of the IL-6 were significantly increased in AD mice upon JA845 intervention.

### Effect of *B. coagulans* JA845 on Regulation of MyD88/TRAF6/NF-κB and Nrf2/HO-1 Signaling Pathway

**Fig. 4:**
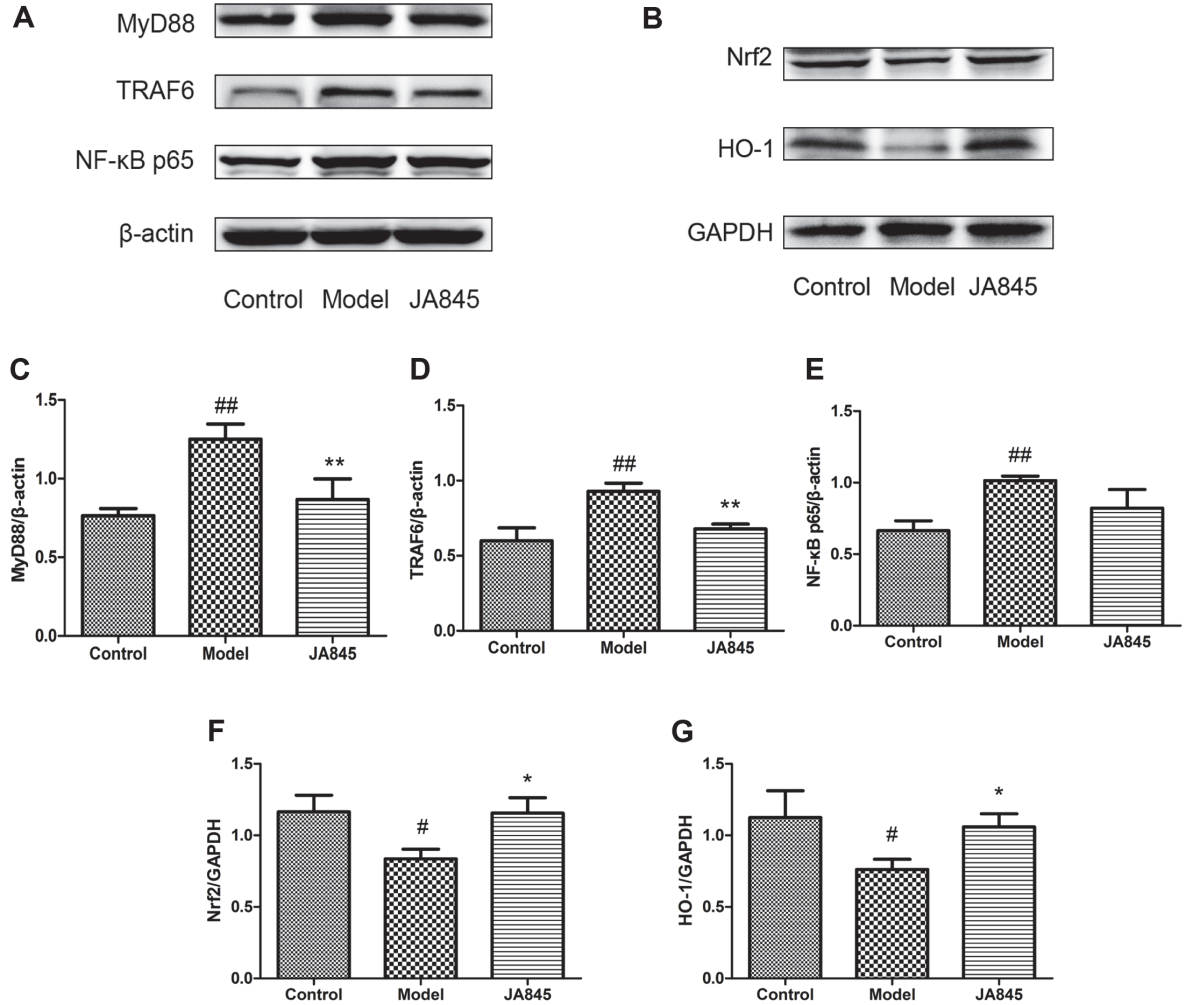
*B. coagulans* JA845 regulated MyD88/TRAF6/NF-κB and Nrf2/HO-1 signaling pathway in D-gal/AlCl_3_-induced AD model mice. (**A**) MyD88, TRAF6, NF-κB p65 protein expressions in brain of mice. (**B**)Nrf2, HO-1 protein expressions in brain of mice. (**C-G**) Quantitative analysis of immunoblot bands. *p < 0.05, **p < 0.01, ***p < 0.001, compared with model group. #*p* < 0.05, ##*p* < 0.01, ###*p* < 0.001, compared with control group.

We further explored whether the MyD88/TRAF6/NF-κB and Nrf2/HO-1 signaling pathways were involved in the beneficial effects of JA845 on anti-inflammation and antioxidation in AD model mice. As shown in Fig. 4, D-gal/AlCl_3_ stimulation contributed to a dramatic increase in the expression levels of MyD88, TRAF6 and NF-κB, significant reduction in the expression levels of Nrf2 and HO-1. However, pre-treatment with JA845 markedly inhibited the expression levels of MyD88, TRAF6 and NF-κB, increased he expression levels of Nrf2 and HO-1. The results indicated that MyD88/TRAF6/NF-κB inhibition and Nrf2/HO-1 activation might mediate D-gal/AlCl_3_-induced inflammatory response and oxidative stress.

## Discussion

As the most common neurodegenerative disease, AD is characterized by cognitive decline, extracellular Aβ deposition, intracellular hyperphosphorylated tau forming neurofibrillary tangles, neuronal loss and neuroinflammation. It has been reported that D-gal/AlCl_3_ stimulation could mimic AD-like pathological features and basic pathogenesis, including oxidative stress and pro-inflammatory signals contributing to these pathological features [2, 3]. Interestingly, growing evidences suggested that probiotics have beneficial effects on the delay of AD progression [16]. In our study, we showed that JA845 administration prevented D-gal/AlCl_3_-induced cognitive decline and AD-like pathological features, with a significant reduction of oxidative stress and inflammation.

Cognitive decline, including memory impairment and behavioral abnormalities, which commonly occur in the early stage of AD, might be accompanied by lesion of the hippocampus and entorhinal cortex [17]. It has been reported that long-term D-gal/AlCl_3_-exposure contributes to AD-like symptoms, and consistent with previous research [18], D-gal/AlCl_3_ stimulation resulted in poor behavioral performance and neuronal loss in AD group. Behavioral test and H&E staining indicated that JA845 pre-treatment markedly alleviated D-gal/AlCl_3_-induced cognitive deficits and lesion of hippocampus. Additionally, Aβ accumulation and tau pathology were also found after D-gal/AlCl_3_ exposure. As a neuropathological hallmark of AD, Aβ accumulation has been identified in the brains of AD patients, and might be responsible for cognitive deficits. Hyperphosphorylated tau forming neurofibrillary tangles are related to the severity and duration of dementia [19]. In our study, we found that JA845 intervention attenuated Aβ accumulation and tau pathology in AD model mice, implying positive effects of JA845 in delay of AD progression.

Although the amyloid and tau hypothesis plays a critical role in AD pathogenesis, it is not restricted to this. Aβ deposits and neurofibrillary tangles induced neurotoxicity, resulting in synaptic impairment and oxidative damage, which is widely believed to be responsible for the underlying neurodegenerative mechanism of AD [20]. The decreased antioxidant enzymes activities and increased levels of oxidative markers were observed in D-gal/AlCl_3_-induced AD model mice, in agreement with previous study [21]. We found that JA845 pre-treatment significantly increased SOD, CAT and GSH-Px activities, and decreased the serum levels of MDA and NO. Additionally, misfolded and aggregated Aβ simultaneously initiated the innate immune response characterized by secretion of inflammatory cytokines, which accelerated AD development and severity [3]. It has been reported that inactivated *B. coagulans* GBI-30, 6086 stimulated immune response and increased the levels of anti-inflammatory cytokines and chemokines, resulting in the anti-inflammatory action [22]. A clinical study suggested that long-term supplementation of *B. coagulans* BC30 could modify gut microbiota composition and potentially increase secretion of anti-inflammatory cytokines in the elderly (aged 65-80 years) [23]. In our study, JA845 administration significantly reduced serum levels of pro-inflammatory cytokines TNF-α and IL-1β, and increased those of IL-6 in AD model. Based on these results, we demonstrated that JA845 pre-treatment not only has potential in preventing oxidative stress but also attenuating neuroinflammation.

The myeloid differentiation primary response gene 88 (MyD88), as an adaptor protein recruited by Toll-like and interleukin receptor, contributes to secretion of inflammatory cytokines and initiates inflammatory responses through activating MyD88-dependent pathway [24]. Previous studies suggested that MyD88 deficiency in AD model mice not only ameliorated cognitive dysfunction, but also reduced Aβ deposits and inflammatory activation [25, 26]. In this study, D-gal/AlCl_3_ exposure significantly increased the expression of MyD88, TRAF6 and NF-κB, whereas JA845 pre-treatment effectively inhibited the expression of MyD88, TRAF6, NF-κB in AD model mice, which suggested that the anti-inflammatory and neuroprotective effects of JA845 on D-gal/AlCl_3_-induced neuroinflammation are connected with downregulation of MyD88/TRAF6/NF-κB pathway. Conversely, one study reported that the mice with loss of the MyD88 protein showed impaired spatial and working memory [27]. Contradictions between the two sets of data have not been clarified and might be related to differences in testing methods or experimental animals (D-gal/AlCl_3_-induced model mice vs. MyD88-deficient mice).

Activation of the nuclear factor E2-related factor 2 (Nrf2), as the master regulator for maintaining oxidative homeostasis, elicits a neuroprotective effect in neurodegenerative diseases [28]. The reduced expression of nuclear Nrf2 was observed in brain regions of AD cases [29]. Additionally, it has been reported that absence of Nrf2 activity exacerbates the AD pathological process [30]. The activated Nrf2 mediated the expression of phase II enzyme HO-1, which elevated the capacity of antioxidant and protected cells from oxidative injury [31]. Consistently, we observed that the nucleus expression of Nrf2 increased after preventive supplement of *B. coagulans* JA845 for 10 weeks. Moreover, the decreased expression of HO-1 was found in AD model mice, whereas prophylactical administration of JA845 elevated expression of HO-1, implying that JA845 elicited antioxidative effect through regulation of Nrf2/HO-1 pathway.

As we know, the beneficial effect of probiotics intervention is mostly completed by regulating gut microbiota, and *B. coagulans* is no exception. *B. coagulans* is unique in that spores can germinate and colonize the intestinal tract, which directly normalizes the gut microbiota or produces bacterial metabolites to exert its probiotic effect. *B. coagulans* JA845 was demonstrated as having anti-inflammatory and antioxidant effects on D-gal/AlCl_3_-induced AD symptoms in the current study; however, it remains a matter of conjecture whether the underlying mechanisms are associated with modulation of gut microbiota and production of metabolites. Further study is needed to explore the interaction between the beneficial effects of *B. coagulans* and changes of intestinal flora.

In conclusion, this is the first investigation of *B. coagulans* JA845 as a potential probiotic strain exhibiting efficacy on the prevention of D-gal/AlCl_3_-induced AD symptoms. The findings of the study indicate that *B. coagulans* JA845 pre-treatment not only prevented cognitive decline but also alleviated neuropathological alterations, particularly hippocampal lesion, Aβ accumulation and tau pathology. Moreover, *B. coagulans* JA845 attenuated oxidative stress and inflammatory response through regulation of Nrf2/HO-1 and MyD88/TRAF6/NF-κB pathway. These results manifest *Bacillus coagulans* JA845 as a suitable candidate with therapeutic potential in AD.
